# Density-Dependence in Space and Time: Opposite Synchronous Variations in Population Distribution and Body Condition in the Baltic Sea Sprat (*Sprattus sprattus*) over Three Decades

**DOI:** 10.1371/journal.pone.0092278

**Published:** 2014-04-03

**Authors:** Michele Casini, Tristan Rouyer, Valerio Bartolino, Niklas Larson, Włodzimierz Grygiel

**Affiliations:** 1 Swedish University of Agricultural Sciences, Department of Aquatic Resources, Institute of Marine Research, Lysekil, Sweden; 2 University of Oslo, Centre for Ecological and Evolutionary Synthesis (CEES), Department of Biology, Oslo, Norway; 3 UMR 212 EME, IFREMER (Institut Français de Recherche pour l'Exploitation de la mer), Sète, France; 4 University of Gothenburg, Department of Earth Sciences, Gothenburg, Sweden; 5 National Marine Fisheries Research Institute (NMFRI), Department of Fisheries Resources, Gdynia, Poland; Dauphin Island Sea Lab, United States of America

## Abstract

Spatio-temporal density-dependent processes are crucial regulatory factors for natural populations. However, there is a lack of studies addressing spatial density-dependence in fish growth. A previous investigation has suggested spatio-temporal density-dependence in body condition of Baltic sprat. Here, we used different techniques, such as centre of gravity, distance, and homogeneity indices, to better characterize the spatial and temporal variations in sprat density and body condition in the Baltic Proper. Our results evidenced a negative spatio-temporal co-variation between the centres of gravity of density and maximum condition. In the 1980s-early 1990s both centres were located in the middle of the Baltic Proper. From the mid 1990s the centres progressively separated in space, as the sprat population moved towards the north-eastern Baltic Proper, and the centre of maximum condition towards the south-western areas. Moreover, at low abundances, sprat density and condition were homogeneously distributed in space, whereas at high abundances both density and condition showed pronounced geographical gradients. The ecological processes potentially explaining the observed patterns were discussed in the light of the Ideal Free Distribution theory. We provide evidence that the shift in the spatial distribution of cod, the main predator of sprat, has been the main factor triggering the overall spatial changes in sprat density, and thus condition, during the past thirty years. The spatial indices shown here, synthesizing the spatio-temporal patterns of fish distribution, can support the implementation of the EU Marine Strategy Framework Directive.

## Introduction

The spatial distribution of natural populations is determined by factors such as intrinsic population characteristics, trophic interactions, hydro-climatic features and/or anthropogenic perturbations. In marine environments, a number of studies have evidenced the effect of population abundance on the spatial variation of several demographic and life-history traits, such as dispersal or migration [1], survival [2], [3] and reproductive success [4]. Individual growth is one of the key life-history traits that determine the fitness of an individual or population, and therefore spatial changes in growth may have substantial implications for population dynamics. However, to our knowledge, density-dependence in growth of natural fish populations over space has not received much attention. Theoretically, if individuals from a population are capable to move freely across space and choose the most suitable habitat available, they should distribute so that their fitness would be maximised and homogeneous throughout their range of distribution (“ideal free distribution”, IFD [5]). Studying departures from this expectation has always been seen as an opportunity to shed light on the processes leading to changes in the spatial distribution of fish populations [6]. Direct measures of fitness or habitat suitability are rarely available from the field. However, spatial variation in fish individual growth and condition, as well as other fitness-related life-history traits, has been proposed as alternative reasonable measures to test departures from theoretical patterns [7]. In particular, field measurements of individual growth and condition over long periods of time and large spatial extension are relatively common for numerous well-monitored fish populations. Investigating the spatial patterns in these traits may thus be used to make inference on the mechanisms behind density-dependence.

In fisheries science, spatial considerations in population dynamics have started to receive the due attention only very recently [8]. However, the importance of accounting for both spatial and temporal scale in the study of marine populations is recognized [9]–[10]. Spatial investigations, including the study of spatial density-dependence, are therefore crucial for a sound understanding of fish populations and therefore for the management of exploited resources [8]–[12].

**Figure 1:**
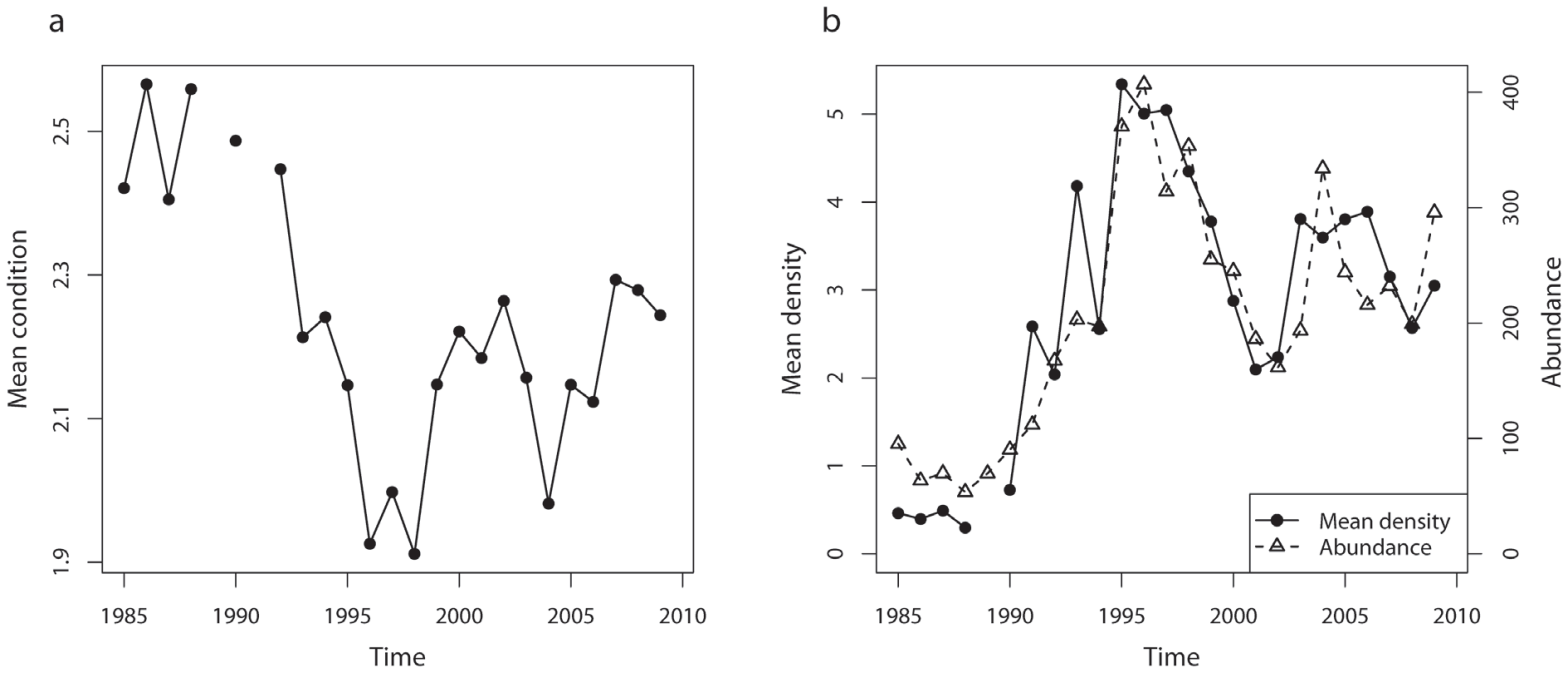
Time-series of sprat mean yearly density and yearly mean condition. The mean density (and condition) is computed averaging over the Baltic Proper the mean density (and condition) from each ICES statistical rectangles (relation between mean density and mean condition: Spearman's C = −0.89, p = 0.0006). The abundance corresponds to the total numbers of sprat (ages 1+) from stock assessment estimates [13].

The abundance of the Baltic sprat (*Sprattus sprattus*) population has increased during the past thirty years [13] as a consequence of the drop in its main predator cod (*Gadus morhua*) and raised temperature [14], [15]. The increased sprat abundance caused in turn a density-dependent temporal decrease in individual condition [16]–[18] (Figure 1). The sprat is able to depress by predation the biomass of its main zooplankton preys [14], suggesting feeding competition as the most likely process behind the density-dependent response in condition [18]. The Baltic Sea has therefore shown during the past thirty years the occurrence of a trophic cascade, from the piscivore cod down to zooplankton, which has affected the annual condition of the zooplanktivorous sprat via density-dependence [14], [18]. Spatial density-dependence in condition has also been suggested for the Baltic sprat [19]. However, no investigation of the temporal changes in either the strength of the spatial density-dependence or in the patchiness of sprat demographic variables has been performed, which could provide a first insight into the forces originally triggering such a response.

Here we used a unique, extensive dataset of biological data on sprat collected in the Baltic Sea during autumn international acoustic surveys in 1985–2009. Using descriptive spatial statistics, our objectives were 1) to investigate the temporal changes in the distribution of the sprat population and its body condition as indicator of spatial density-dependence, 2) to investigate whether the strength of the spatial density-dependence varied in time, and 3) to investigate whether the patchiness of the sprat distribution and condition varied in time. We also discussed possible explanations for the observed spatial patterns in light of the current knowledge on the Baltic hydrology and predator-prey interactions.

## Material and Methods

### Data

#### Sprat densities

Time-series of sprat abundance (total number, ages 1+) by ICES statistical rectangle (0.5° in latitude and 1° in longitude) were extracted from the ICES BAD1 international database [20] and survey reports stored at the former Swedish Board of Fisheries, Institute of Marine Research [19]. These data are based on hydroacoustic surveys regularly performed in September–October in the open areas of the central Baltic Sea (the Baltic Proper, corresponding to the ICES Subdivisions (SDs) 25–29) and are regularly used as tuning indices for the assessment of the Baltic sprat stock [13]. Sprat density (number/squared nautical mile) was calculated yearly for the whole survey area (mean yearly density), and for each ICES rectangle, for the period 1985–2009. Acoustic data prior to the 1985 are not available at the resolution of the ICES rectangle, and thus could not be used in the analyses.

#### Fish sampling and condition computation

Sprat individuals were collected during the hydroacoustic surveys using mid-water trawls in the pelagic zone and near the bottom layers, depending on the fish vertical distribution detected by the echosounder (see [20] for details on fishing operation and sampling procedure). Prior to the analysis, fish body weights were rounded to the nearest 1 g, and the total lengths were rounded down to the nearest 0.5 cm.

For the estimation of fish condition, we used the Fulton's K = TW/TL^b^, where TW and TL are respectively the total weight and total length of each fish, and *b* is the slope of the overall ln (TL) – ln (TW) relationship. Condition was estimated yearly for the whole survey area (mean yearly condition) and for each ICES statistical rectangle using the specimens between 120 and 130 mm total length, which corresponds to the majority of the lengths sampled during the survey and present in all years and ICES rectangles. Sprat of this size has most likely already reached maturity [21].

Previous investigations have shown for clupeids (e.g., Baltic sprat and spring-spawning Atlantic herring) that the seasonal variations in morphometric condition indices, calculated based on fish weight and length, follow the seasonal changes in the lipid content of their muscles [22], [23]. Therefore, the condition computed in our study reflects the fat and energy content of the fish.

### Statistical analysis

#### Centres of gravity

The centres of gravity for density and condition were obtained by computing the average of latitudes and longitudes, weighted by densities and condition, respectively [24], [25]. In order to focus the analysis on the spatial patterns of the maximum density and maximum condition, the centres of gravity were obtained using data above the median of the density and condition distributions, respectively. The robustness of the approach was ensured by the following process. The years with too few points available over space were excluded from the analysis in order to preserve a consistent spatial coverage. Then, the centres of gravity were estimated by a bootstrap procedure. For each year and each variable (density and condition), the centres of gravity were computed on the whole spatial data set minus one data value. Removing sequentially every data value on the map allowed for constructing a statistical distribution for the centres of gravity and an associated confidence interval. The estimates for the centre of gravity coordinates were obtained from the median of each yearly statistical distribution. The time series of coordinates obtained for the density were then plotted against those for condition to investigate the link between their trajectories. This approach allowed summarizing the patterns of density and condition over space and to follow their evolution over time. Under the effect of limited available resource we may expect a negative spatial density-dependence between density and condition, which would result in centres of gravity moving in opposite directions.

#### Correlations over space

In order to interpret the movement of the centres of gravity over space, and to explore the intensity of the relationship between density and condition over time, the following approach was used. For each year, the densities were correlated to the condition values over space (based on the ICES rectangles). Thereafter, the strength of the spatial correlations between density and condition was compared to the distance between their centres of gravity, the mean yearly density and mean yearly condition.

#### Spatial patchiness

The patchiness of the distributions for density and condition was investigated over time. The spatial autocorrelation was used as a proxy for patchinesss, as high values are associated to larger patches over the considered space [26], [27]. To do so, the Moran index (I) was calculated for each yearly map of sprat densities and condition, using the function implemented in the R package “ape” [28]:
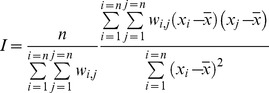
with *n* the number of observations, *x* the observations, and *w* the distance weights between observations calculated as:
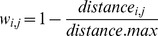
with *distance_i,j_* the Euclidian distance between observations *i* and *j* and *distance.max*, the maximum distance observed.

The Moran I measures spatial autocorrelation, and evaluates whether the pattern expressed is clustered or dispersed. Positive values (towards 1) indicate clustering, while negative values (towards −1) indicate dispersion. The Moran I allowed also to check whether a centre of gravity located in the middle of the study area was related to the presence of a real patch in that location or due to a lack of spatial gradients in the seascape.

The significance of the Spearman's correlation coefficients was controlled for autocorrelation by adjusting the numbers of degrees of freedom [29].

All computations were performed using R (version 2.12.0, R Development Core Team, 2010; available at http://www.R-project.org).

## Results

### Statistical analysis

#### Centres of gravity

The changes in sprat mean yearly density and mean yearly condition from the hydroacoustic surveys are shown in Figure 1. The mean yearly density was strongly correlated to the total sprat abundance in the Baltic Sea from analytical stock assessment [13].

**Figure 2:**
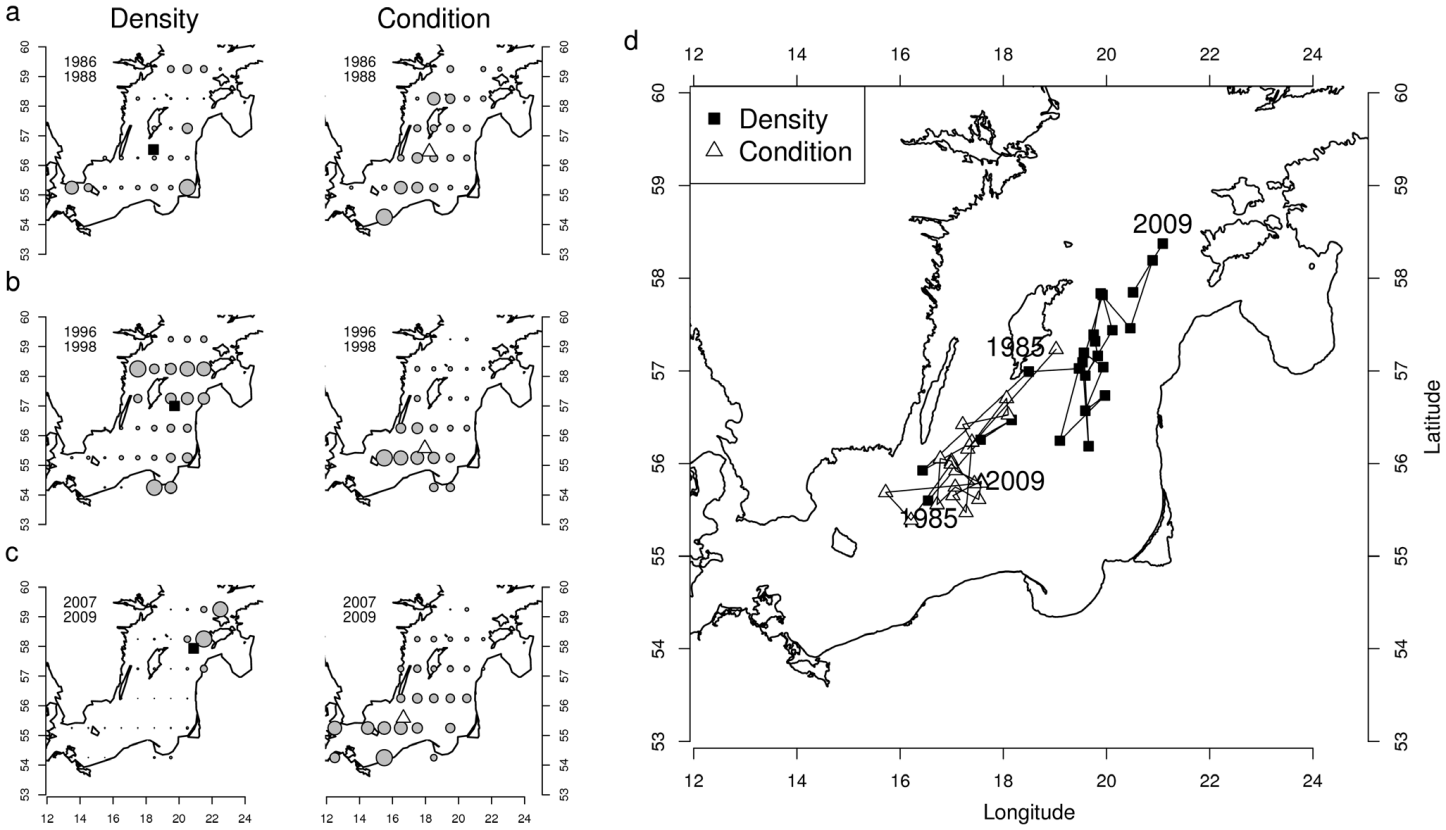
Temporal evolution of the centres of gravity for sprat density and condition. (a), (b) and (c) Maps illustrating the centres of gravity for the periods 1986–1988, 1996–1998 and 2007–2009. The bubble size is proportional to the relative density (black squares) and condition (white triangles) over the maps; (d) Temporal trajectories of the centres of gravity over the Baltic Proper summarized on the map.

The centres of gravity of density and condition displayed large spatial changes over time (Figure 2). In the 1980s, both centres of gravity were generally located in the middle of the Baltic Proper (Figure 2a). In the 1990s, the centre of gravity of density moved towards the north-east, whereas the centre of gravity of condition moved towards the south-west (Figure 2b), a pattern that persisted and reinforced in the 2000s (Figure 2c). Starting from relatively similar locations in the heart of the Baltic Proper, the trajectories of the centres of gravity diverged in opposite directions over time, suggesting a negative relationship between the spatial patterns (Figure 2d).

**Figure 3:**
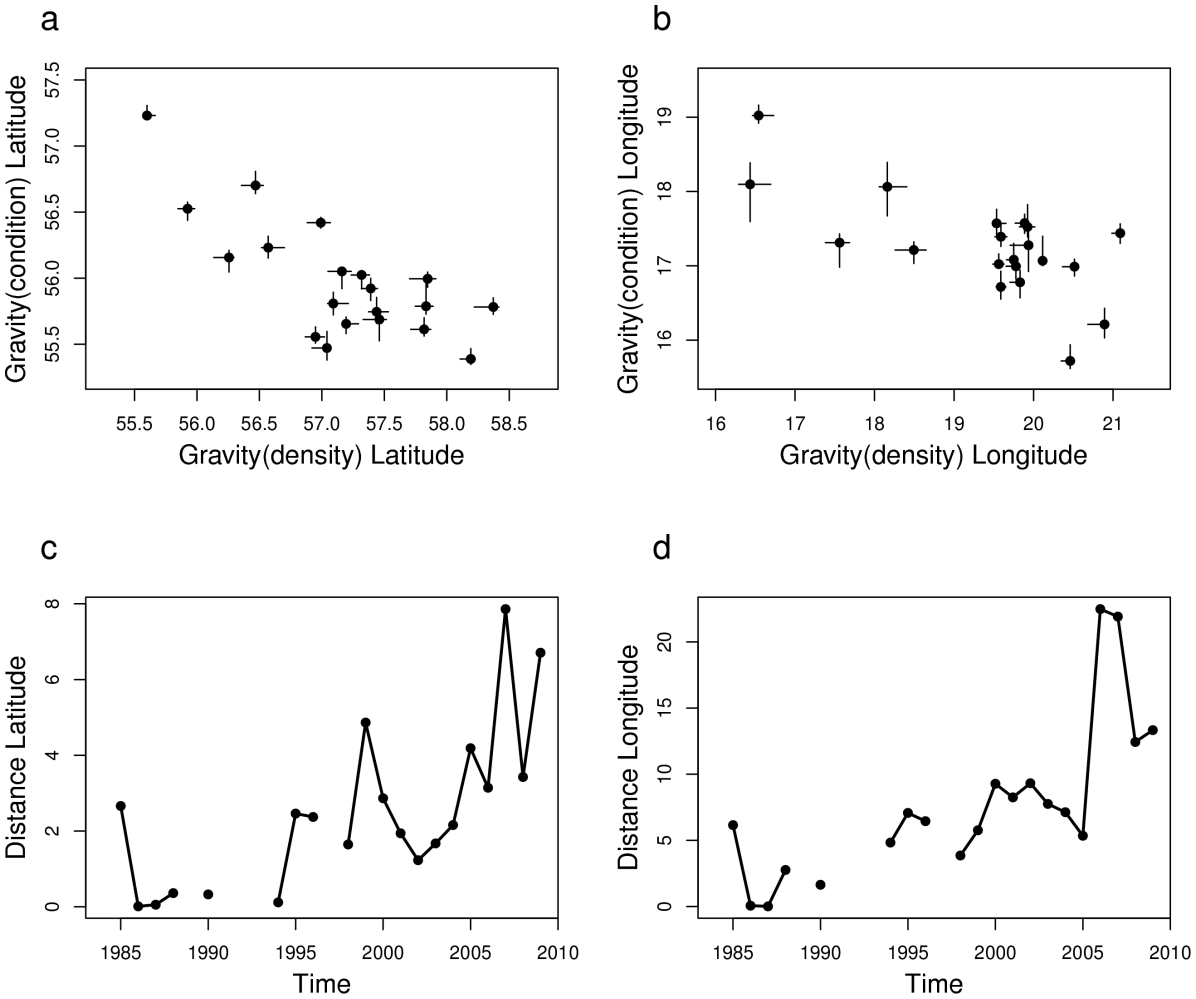
Spatial relation between the centres of gravity for sprat density and condition. (a) and (b) relation between the positions of the two centres of gravity, in latitude and longitude (whiskers indicate the confidence interval at 95%); (c) and (d) temporal evolution of the distance between the latitudes and the longitudes of the centres of gravity.

The times series of coordinates of the two centres of gravity displayed a significant negative association over latitudes (Spearman's C = −0.62, p = 0.02) and longitudes (Spearman's C = −0.52, p = 0.04), which confirmed their movement in opposite directions (Figure 3a,b). The distance between the centres of gravity increased over time, both in latitude and longitude (Figure 3c,d). This divergent pattern over time occurred in parallel to an increase in the mean yearly density and a decrease in the mean yearly condition (Figure 1).

#### Correlations over space

**Figure 4:**
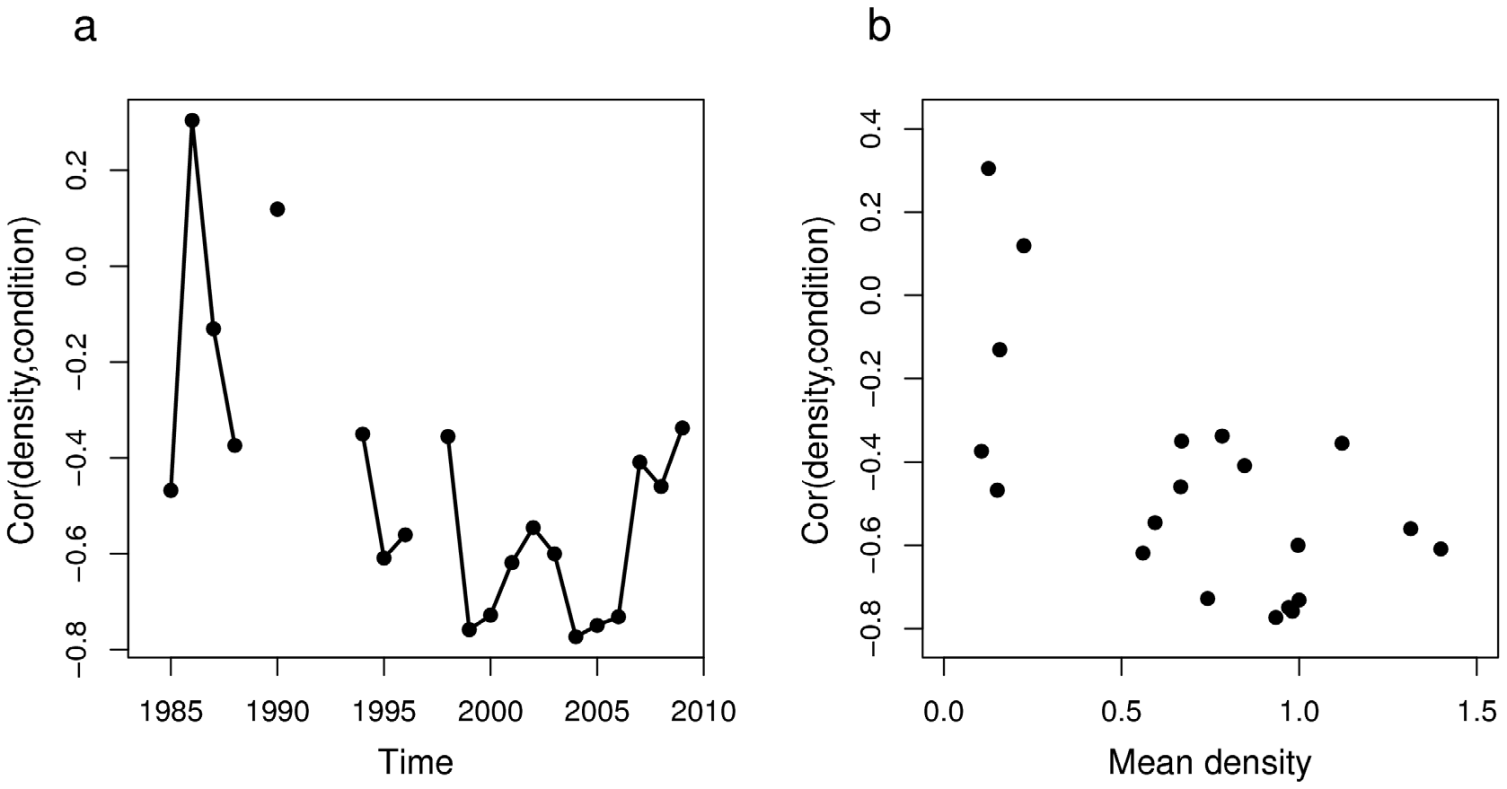
Changes in the correlation between the centres of gravity for sprat density and condition. (a) temporal evolution of the correlation between the centres of gravity for density and condition; (b) correlation between the two centres of gravity as function of mean yearly density.

The spatial correlation between density and condition decreased over time and became increasingly more negative (Figure 4a). This was further confirmed by the negative association between the time series of spatial correlations and the mean yearly density (Spearman's C = −0.53, p = 0.08) (Figure 4b). The higher the mean yearly density (related to annual total abundance), the stronger was the negative relationship between density and condition over space and the further away were the centres of gravity of density and condition.

#### Spatial patchiness

**Figure 5:**
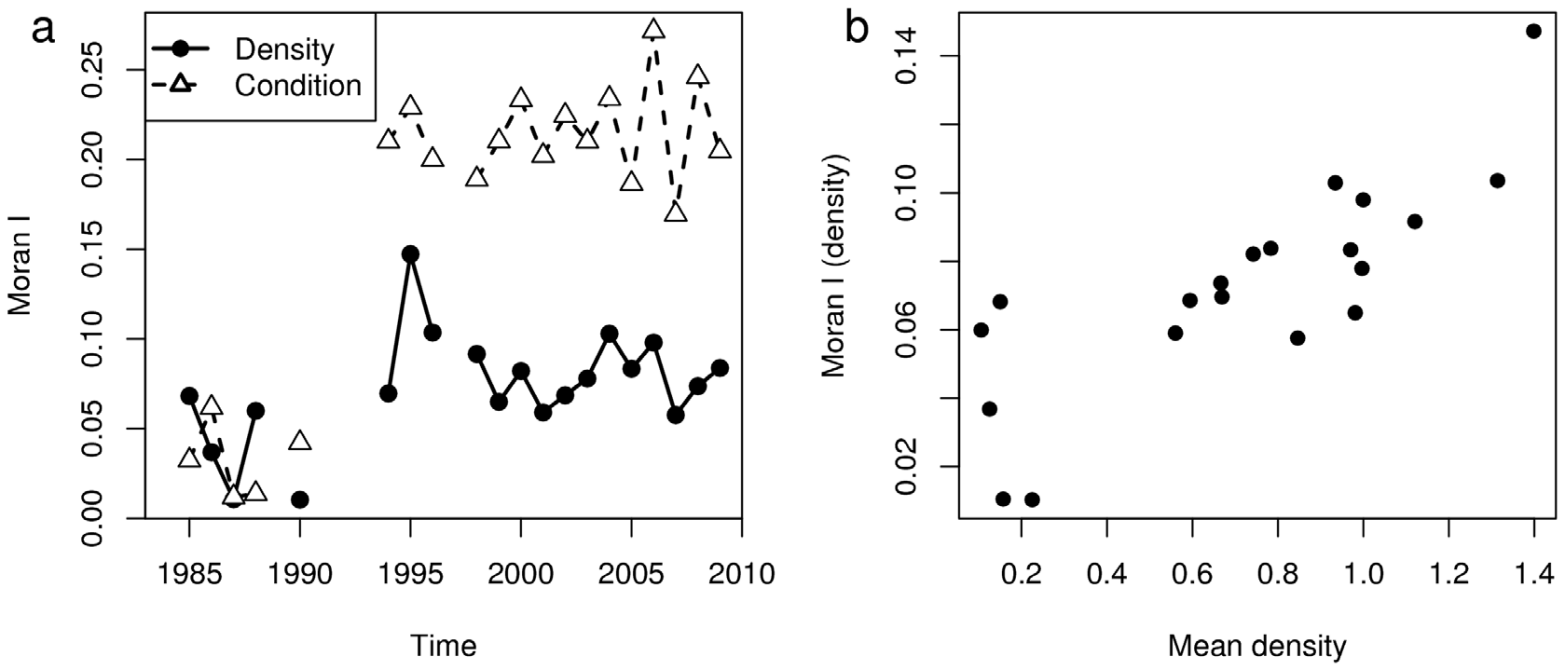
Changes in the Moran Index for the spatial distributions of sprat density and condition. (a) temporal evolution of the Moran Index for density and condition; (b) relation between Moran Index of sprat density and the mean yearly density.

The Moran I was low for both density and condition in the 1980s, and high from the mid 1990s onwards (Figure 5a). This indicated that the spatial autocorrelation among observations (size of the patches) was lower in the 1980s-early 1990s and higher afterwards. Furthermore, the Moran's I computed for density was positively associated to the mean yearly density (Spearman's C = 0.79, p = 0.001), indicating that the higher the density the higher was the spatial autocorrelation of the density (Figure 5b). The low values of Moran's I for density and condition in the 1980s-early 1990s, in combination with centres of gravity located in the middle of the Baltic Proper, suggested homogeneous distributions and a lack of pronounced spatial gradients in density and condition.

## Discussion

In this study we used simple yet powerful spatial techniques, as location and homogeneity indices, to analyse the spatio-temporal relationship between sprat density and body condition in the Baltic Proper. Our results showed a clear-cut spatial inverse variability between sprat density and condition that suggests spatial density-dependence. These findings indicate that the mechanisms driving sprat spatial distribution may play an important role also in shaping density-dependent processes for this species in the Baltic Proper. Although spatial density-dependence for Baltic sprat has been suggested before [19], our study adds new information that provides a first insight into the ecological mechanisms originally triggering such a response. Particularly, we show that the strength of the negative relationship between density and condition varied with sprat abundance, as did the degree of patchiness in the Baltic seascape.

Our study showed that the location of the centre of maximum sprat condition was negatively correlated in space to the centre of the sprat density. In the 1985–1990, the two centres were very close to each other and typically located in the middle part of the Baltic Proper. During this period, both sprat density and condition were also homogeneously distributed within the Baltic Proper, as suggested by the low values in Morans' I. Since the early 1990s, the two centres started to drift apart, with the centre of sprat density progressively shifting towards the north-east and the centre of maximum condition towards south-west. Moreover, the spatial patchiness of both density and condition strengthened since the early 1990s, as indicated by the steep increase in the Morans' I. This illustrates that the sprat population and the values of maximum condition became concentrated in the north-eastern and south-western Baltic Proper, respectively.

The negative spatial relationship between density and condition was generally stronger when the mean population density was higher (i.e. higher sprat abundance). The strengthening of the spatial correlation between density and condition at higher sprat population sizes can be interpreted in two ways that are not mutually exclusive:

1. at high abundances, the population locally approaches the carrying capacity, resulting in reduced individual food intake and condition in the highest density areas;
2. during our study period, the increase in sprat abundance (and mean density) have occurred simultaneously to a contraction of the sprat population into the north-eastern areas producing a stronger spatial gradient in the sprat density and condition. In this case the negative relationship between the mean sprat density and the density-condition spatial relationship would be spurious and related to a third factor, such as for example a predator, driving both sprat abundance and spatial distribution (see below for potential explanations).

Environmentally-driven habitat selection is directly related to the population-level response to the biotic and abiotic factors, which are unequally distributed over space and characterize different habitats. Ultimately, the ecological niche of the population results from the combination of these responses. In a situation of unlimited resources the ecological niche could potentially explain most of a species habitat selection [30]. However, under limited resources habitat suitability is also affected by the density of individuals. As the density increases in a given habitat, its suitability decreases, promoting the movement of individuals towards less suitable low-density habitats. If all individuals are able to freely move across space and choose the most suitable habitat available, fitness-related parameters such as individual growth may be expected to be relatively homogeneous across habitats (Ideal Free Distribution theory, IFD, [5]). In our study, we show that sprat condition was spatially homogeneous only until the early 1990s, whereas thereafter condition started to show a strong spatial gradient. In fact, since the early 1990s the concentration of sprat into the north-eastern Baltic Proper triggered strong density-dependent effects that resulted in a drastic decrease in body condition in this area. In other studies, condition has been related to lipid reserves and therefore to fecundity in Baltic clupeid fish [22], [31]. A low body condition would then also imply a reduced per-capita reproductive output [32]. Consequently, the marked heterogeneities in the spatial distribution of the sprat condition that emerged under high population level may be interpreted as a departure from the optimal use of the habitat as formulated by the IFD.

Three alternative explanations, not mutually exclusive, can be provided to explain the observed departure from theoretical expectations. First, simulation studies showed that under combined temporal and spatial heterogeneity [33], the evolution of dispersal may produce and maintain unequally distributed fitness across the different habitats. The Baltic is characterized by strong environmental gradients, and during the time period investigated it experienced notable changes in the hydrographical spatial patterns [19] that may have affected the spatial distribution of sprat (“abiotic environment-related hypothesis”). However, the centre of sprat density moved towards areas hydrographically disadvantageous for the recruitment of this thermophilic marine species, i.e. areas with lower temperature and lower salinity [34], [35] typical of the northern Baltic Proper. For this reason we consider that this hypothesis does not support the observed patterns.

**Figure 6:**
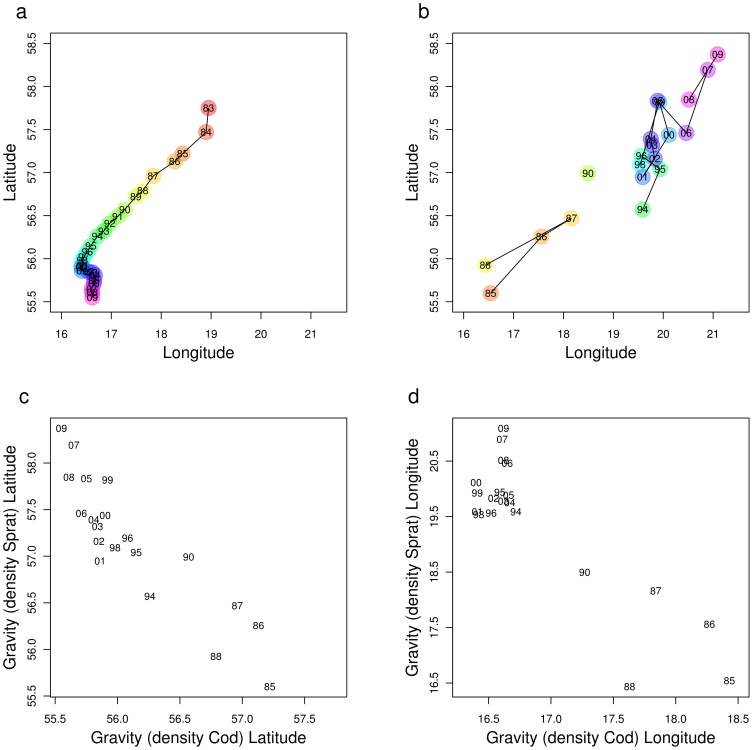
Temporal evolution of the centres of gravity for cod and sprat density. The centre of gravity for cod density was estimated using data on catch/hour from the hydroacoustic surveys regularly performed in September–October in the open areas of the Baltic Proper (ICES Subdivisions 25–29) [36]. The temporal evolution of the centres of gravity for sprat density is the same as presented in Figure 2d. Spearman's C between the latitude of the centres of gravity of cod and sprat = −0.88; Spearman's C between the longitude of the centres of gravity of cod and sprat = −0.50).

**Figure 7:**
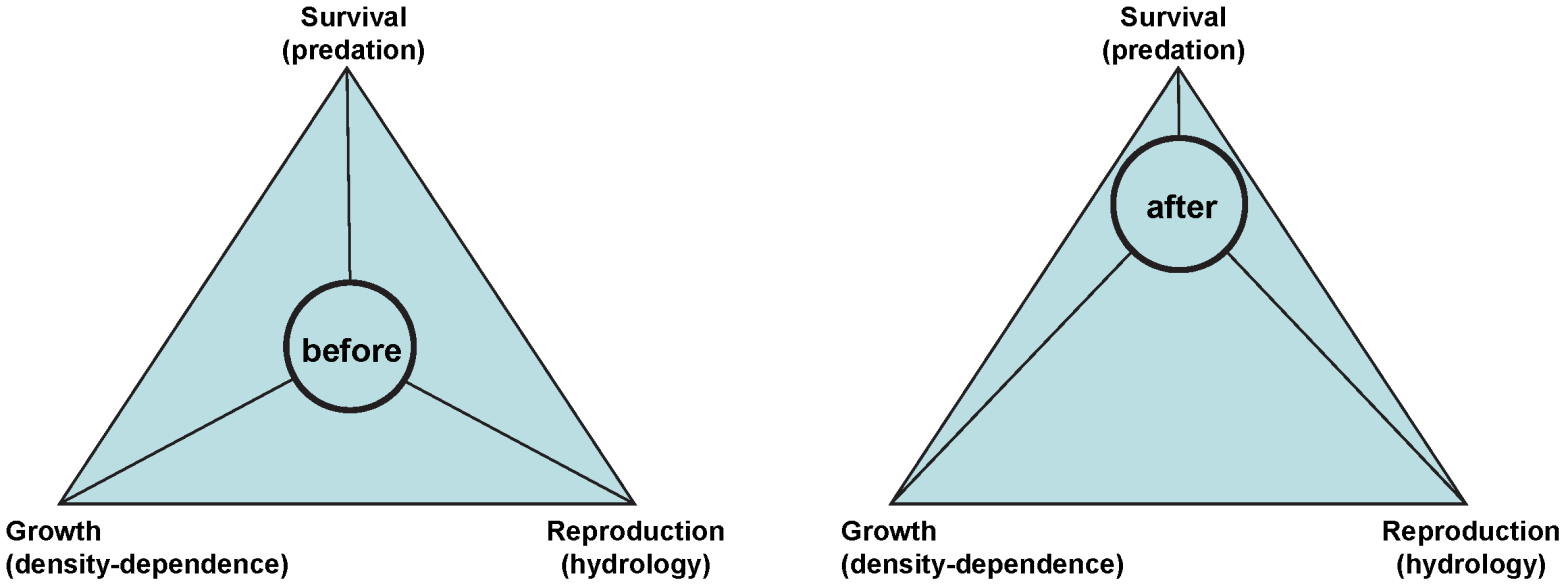
Schematic representation of the fitness of the sprat population before and after the early 1990s. The distance of the circle “sprat” from the angles or the triangles indicates the relative suitability for growth, reproduction and survival (with lower distances indicating more suitable conditions for the corresponding life history traits). In brackets are indicated the processes affecting each life history traits, according to the literature and the current study.

An alternative explanation is that the evolution of habitat selection strategies is expected to be related to the fitness of individuals, for which the measurement of individual condition represents only one facet. Cod (*Gadus morhua*), the main predator of sprat, has drastically decreased in abundance since the late 1980s, while its distribution contracted towards the south-western Baltic Proper [13], [19], [36] (Figure 6). In our case, the sprat individuals after the early 1990s could have actively selected the north-eastern Baltic Proper, an area which had become nearly predator-free, maximising their survival. In this case, the spatio-temporal patterns observed in our study would not disagree with the IFD theory, but would only enlighten one aspect of it. The bulk of the sprat population in the north-east would have a lower condition due to density-dependence, reduced reproductive output (due to lower per-capita fecundity, as well as to lower temperature and salinity), but would experience almost nil predation pressure because of the absence of cod. Conversely, individuals still inhabiting the south-west would experience high cod predation but enhanced growth and reproductive potential (“fitness-related hypothesis” Figure 7).

A third explanation is that the change in the spatial distribution of cod observed during the past three decades in the Baltic Proper (Figure 6) may have triggered the establishment of a spatially heterogeneous predator-prey interaction in the region. Under this hypothesis, the displacement of the sprat population towards the north-east after the early 1990s would be a direct response to a decreased mortality due to the release from cod predation, whereas in the south-west cod predation would have still been able to maintain low sprat densities and allow for higher sprat condition (“predation release hypothesis”).

Currently, there is no sufficient information to favour the “fitness-related hypothesis” or the “predation release hypothesis”. In order to understand the mechanisms behind the changes in spatial distribution of the Baltic sprat population, analyses on site-specific recruitment dynamics, predation pressure (including fisheries) and migration patterns should be performed. Specifically, spatially-explicit characterization of the fitness in the different areas can be difficult to derive but would provide the necessary information to link variability in the spatial distribution and productivity of the sprat stock.

How local densities affect individual performance, such as growth, has been shown in the wild for a number of sessile organisms, for example mussels and limpets [37], [38]. However, there are very few examples of this process in pelagic fish populations inhabiting the open water [19], [39]. Our results contribute to the growing body of evidence showing that also spatial processes can play an important role in density-dependence and population regulation in the wild. In our investigation we found that variations in the spatial distribution of a pelagic fish population have important consequences on how density-dependent processes are shaped in space and maintained in time. The temporal and spatial variability in density-dependent processes are fundamental aspects for the dynamics of fish populations, and understanding their causality is crucial for the development and implementation of a sounder management of the resources [40], [41]. The temporal evolution of the spatial indices as shown in our analyses, by tracking and synthesizing the overall changes in the distribution of Baltic fish populations, can support the implementation of the EU Marine Strategy Framework Directive [42] for the Baltic Sea region.
